# Supplementary material for: Visual features as stepping stones toward semantics: Explaining object similarity in IT and perception with non-negative least squares
Source: Neuropsychologia. 2016 Mar;83:201–26. doi: 10.1016/j.neuropsychologia.2015.10.023 (PMC4783588; doi:10.1016/j.neuropsychologia.2015.10.023)
Supplement: Supplementary file 1 — Supplementary material [file mmc1.pdf]

Visual features as stepping stones toward semantics: Explaining object similarity in IT and perception with non-negative least squares

Kamila M. Jozwik, Nikolaus Kriegeskorte, Marieke Mur

Supplementary Materials

Supplementary Figure 1
Stimuli

Supplementary Figure 2
Model performance for IT (including similarity judgments as model)

Supplementary Figure 3
Model performance for EVC (including similarity judgments as model)

Supplementary Figure 4
Model performance for similarity judgments (including IT as model)

Supplementary Figure 5
Multidimensional scaling of models and single-subject data RDMs

Supplementary Figure 6
Model dimensions relevant for explaining the IT object representation and similarity judgments (each dimension is considered in isolation)

Supplementary Figure 7
Model dimension weights obtained with non-negative least-squares fitting
(dimensions are considered simultaneously)

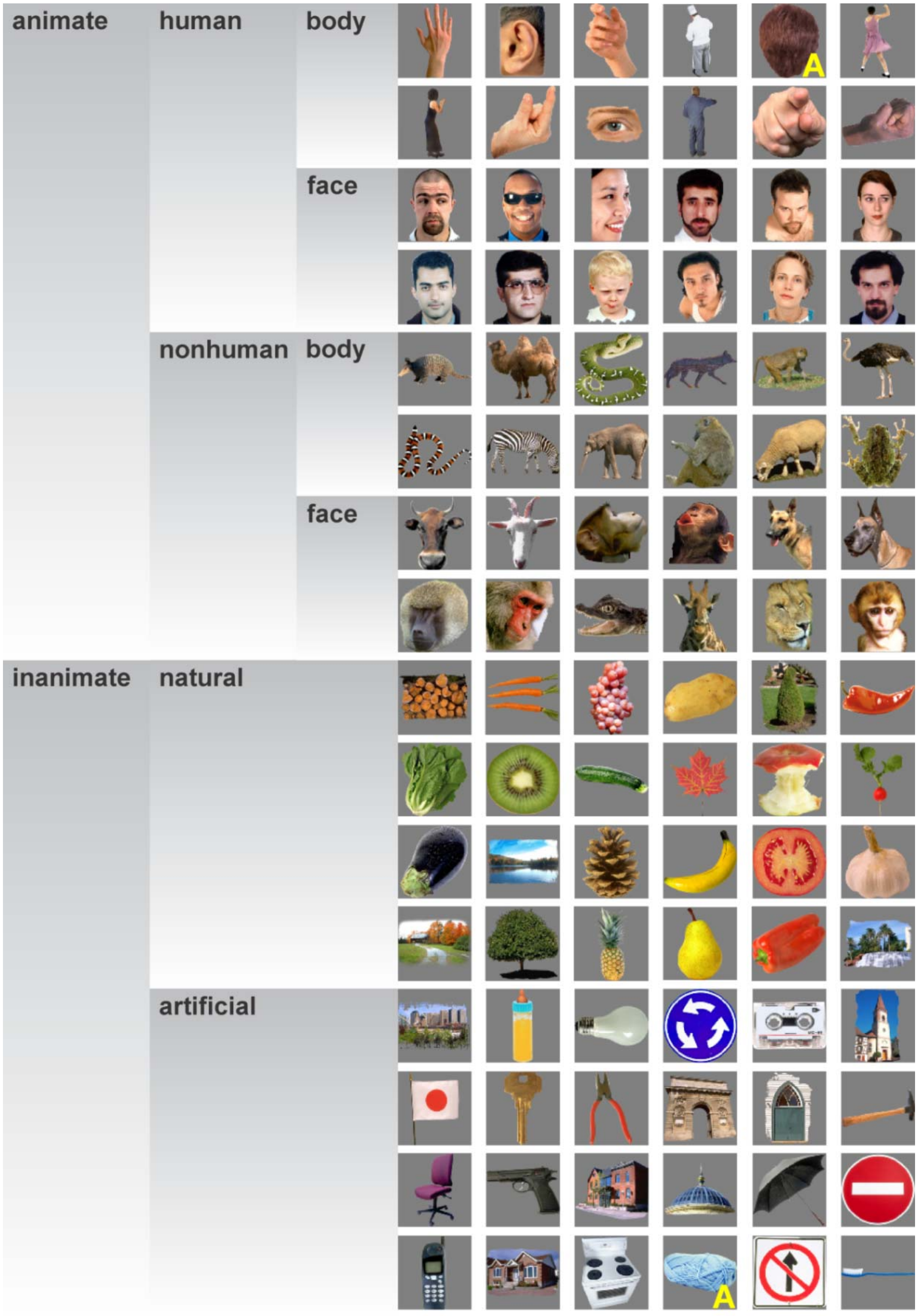

Supplementary Figure 1 | Stimuli. The stimuli marked with an “A” were described as ambiguous by several of the similarity-judgment subjects during debriefing. The figure is adopted from Mur et al. (2013).

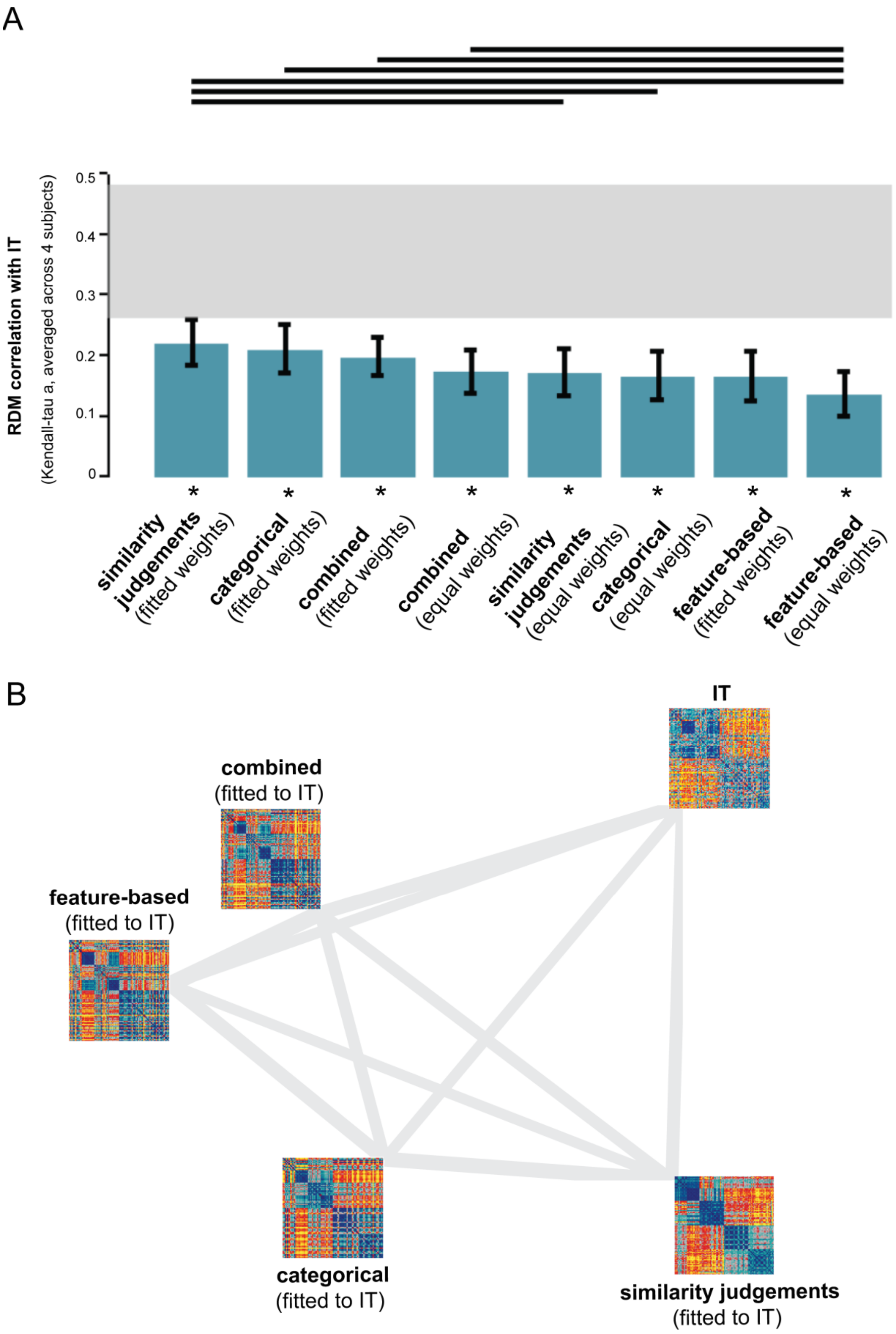

Supplementary Figure 2 | Model performance for IT: similarity judgments perform equally well as the categorical and feature-based model. Same conventions as in Figure 7.

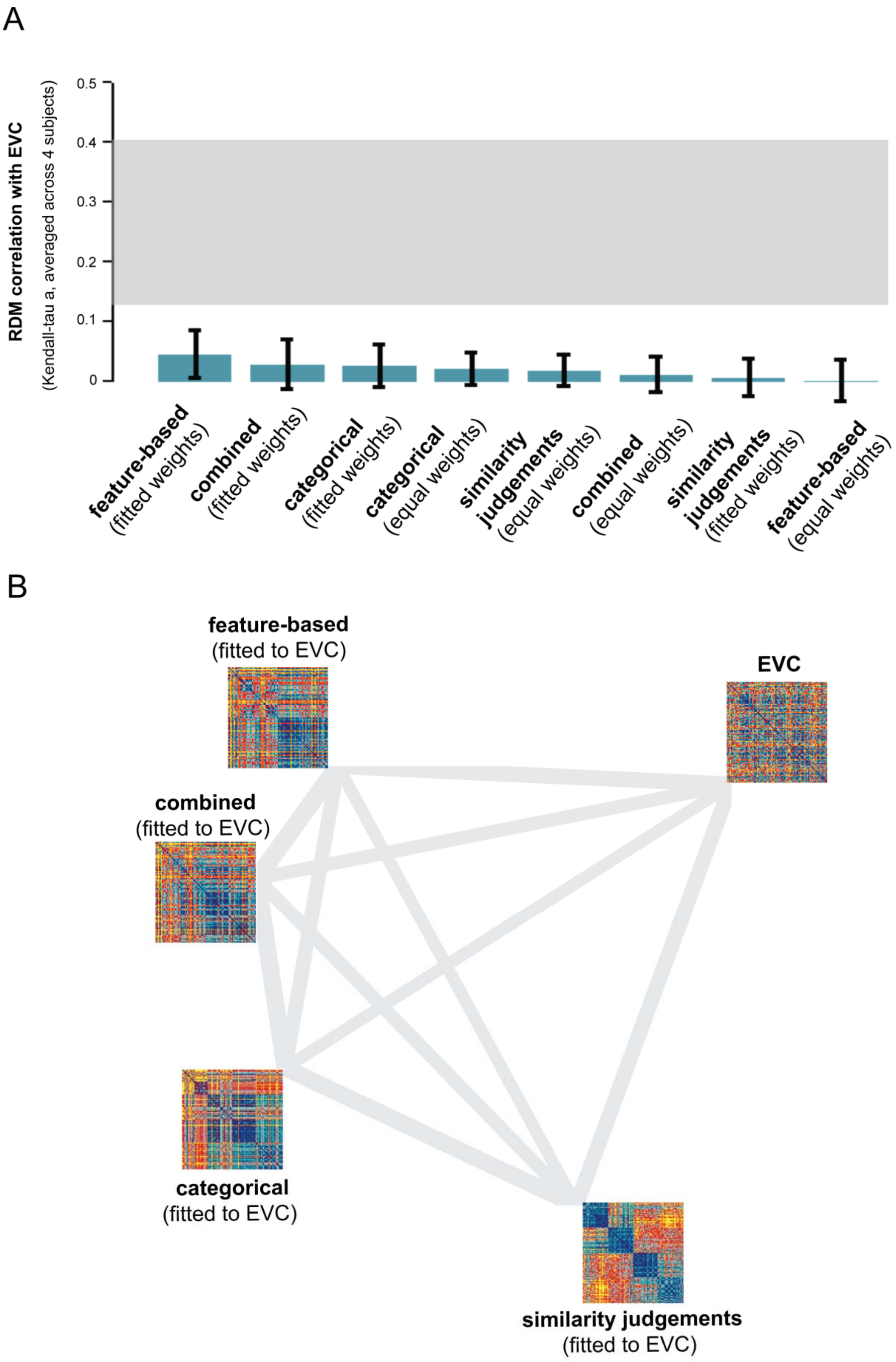

Supplementary Figure 3 | Model performance for EVC: none of the models, including similarity judgments, can explain the EVC representation. Same conventions as in Figure 9.

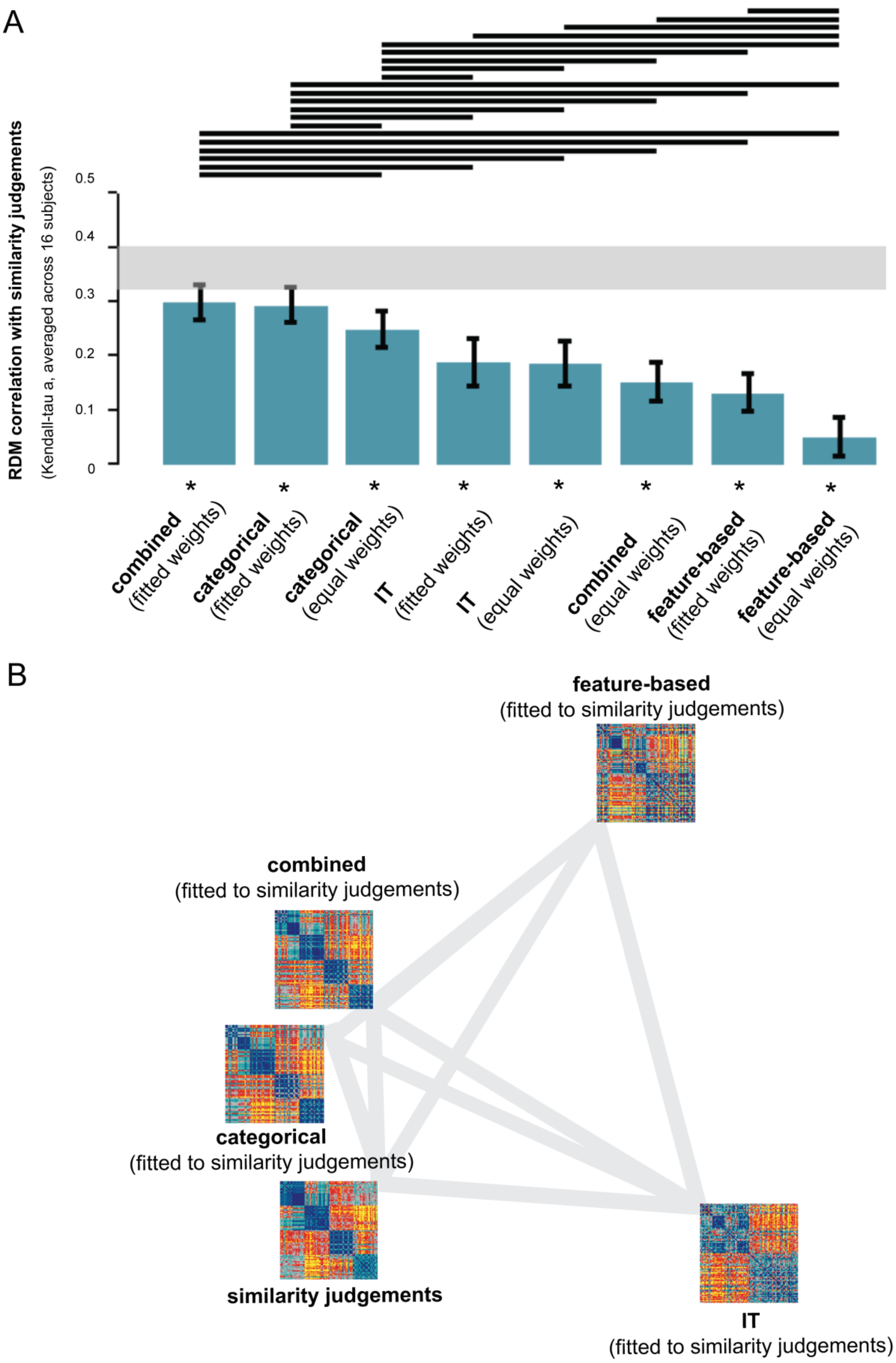

Supplementary Figure 4 | Model performance for similarity judgments: the categorical model outperforms both IT and the feature-based model. Same conventions as in Figure 11.

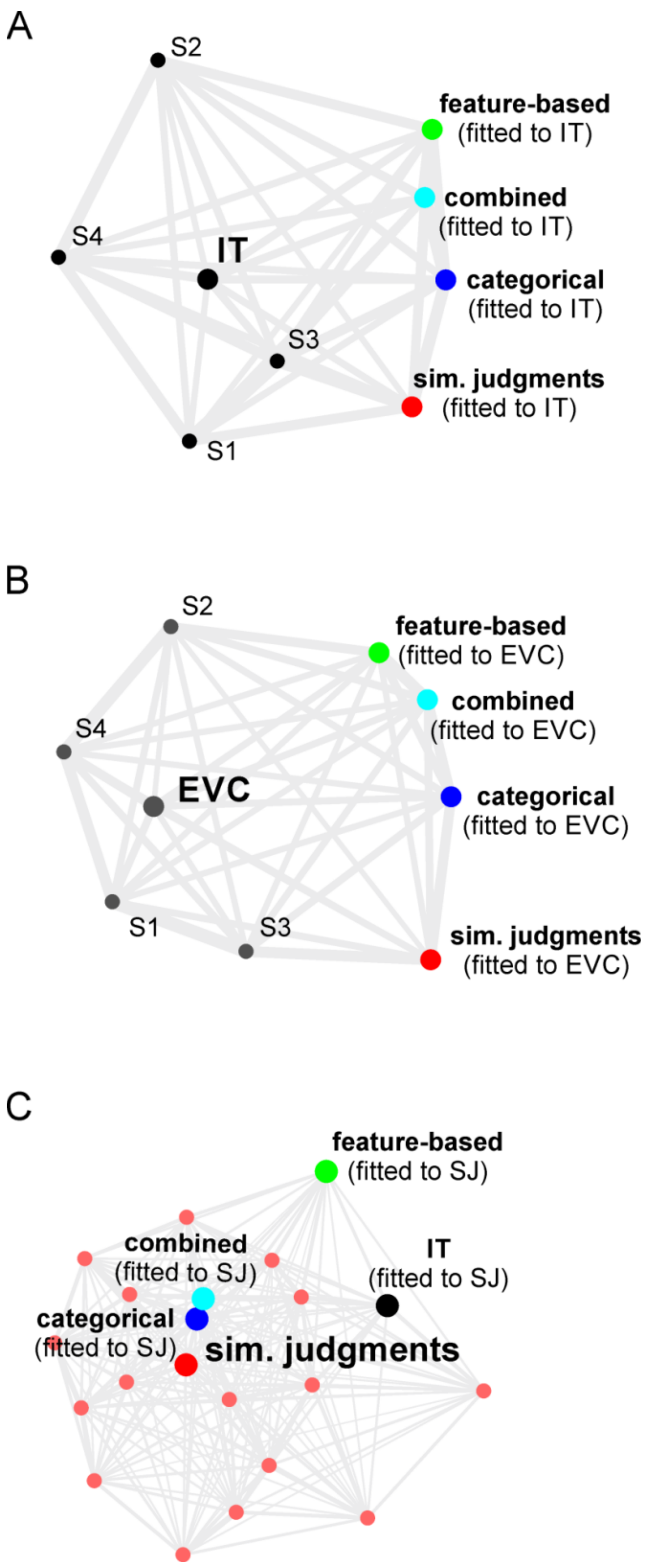

A
S2
feature-based
(fitted to IT)
combined
(fitted to IT)
S4
IT
categorical
(fitted to IT)
S3
sim. judgments
(fitted to IT)
S1
B
S2
feature-based
(fitted to EVC)
combined
(fitted to EVC)
S4
EVC
categorical
(fitted to EVC)
S1
S3
sim. judgments
(fitted to EVC)
C
feature-based
(fitted to SJ)
combined
(fitted to SJ)
IT
(fitted to SJ)
categorical
(fitted to SJ)
sim. judgments

Supplementary Figure 5 | Multidimensional scaling of models and single-subject data RDMs. The multidimensional scaling plots (criterion: metric stress; distance measure: 1-r, where r is Spearman correlation coefficient) visualize the relationships between single-subject data RDMs and model predictions. Small dots indicate single-subject data RDMs (4 subjects for IT and EVC, 16 subjects for the similarity judgments). Distances between RDMs reflect their dissimilarity. The thickness of the lines reflects the inevitable distortions that are introduced by dimensionality reduction. **A** IT **B** EVC **C** Similarity judgments (SJ).

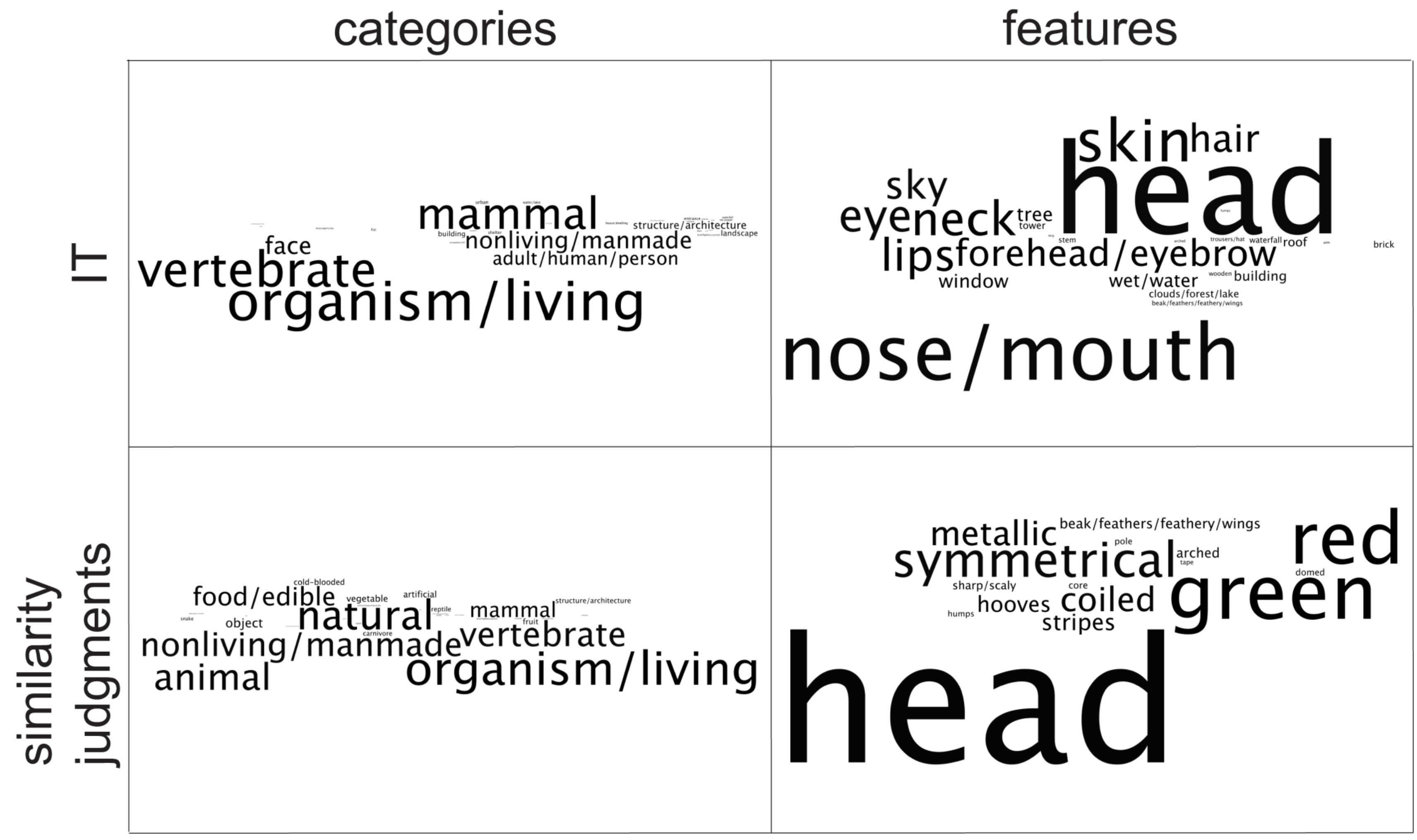
categories
features
IT
similarity judgments
mammal
nonliving/manmade
adult/human/person
structure/architecture
landscape
building
face
vertebrate
organism/living
skin
hair
head
sky
eye
neck
tree
tower
lips
forehead/eyebrow
roof
waterfall
brick
window
wet/water
building
clouds/forest/lake
beak/feathers/feathery/wings
nose/mouth
food/edible
cold-blooded
vegetable
artificial
natural
reptile
mammal
fruit
structure/architecture
object
snake
nonliving/manmade
carnivore
vertebrate
animal
organism/living
metallic
beak/feathers/feathery/wings
red
symmetrical
arched
green
sharp/scaly
hooves
coiled
domed
humps
stripes
head

Supplementary Figure 6 | Model dimensions relevant for explaining the IT object representation and similarity judgments. The word clouds (generated with Wordle) display the category and feature-based labels whose model RDMs show a significant correlation with the IT representation and similarity judgments (stimulus-bootstrap test, $p < 0.05$ corrected). The font size of the category and feature-based labels reflects the relative strength of their correlation with the data dissimilarities.

Supplementary Figure 7 | Model dimension weights obtained with non-negative least-squares fitting. The bar graphs show model dimension weights for each data RDM and model. Only weights for dimensions that applied to more than one image are shown. Each bar represents the mean weight for a model dimension across cross-validation iterations (the number of iterations was around 900 for each model fit). Error bars indicate standard error of the mean. Each bar graph is followed by a page listing the dimension labels. The order of dimension labels follows the order of the ranked bars (e.g. the dimension label on top of the page corresponds to the first bar from the left). SJ refers to similarity judgments.

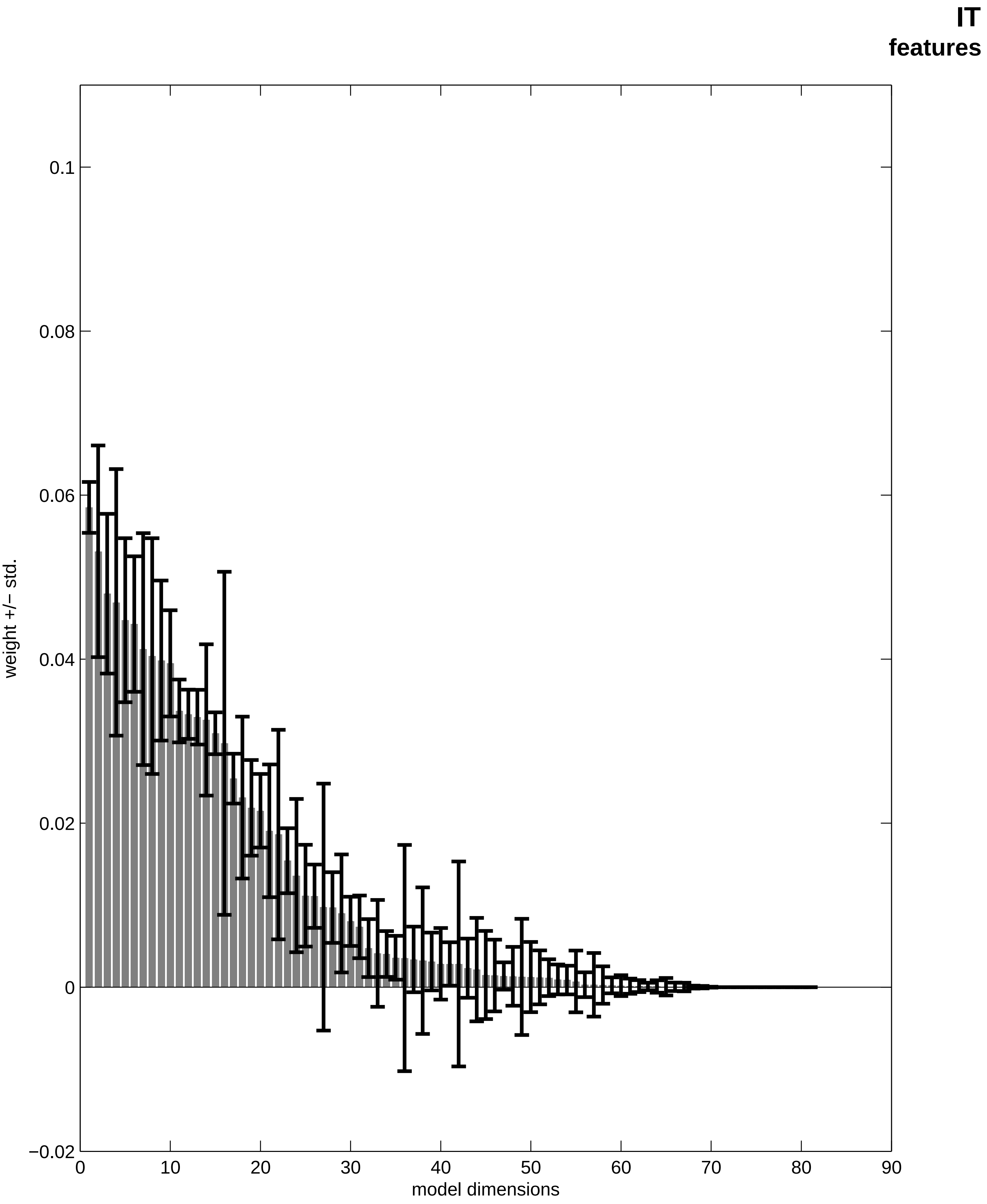

IT
features
weight +/− std.
model dimensions
0.1
0.08
0.06
0.04
0.02
0
−0.02
0
10
20
30
40
50
60
70
80
90

IT
features

head
wooden
woolly
window
building
arrow
handle
hand/fingers
sky
shiny
legs/body
skin
yellow
rounded
nose/mouth
wet/water
plastic
symmetrical
leafy
green
round/circular
wrist
snout
glass
shoulder
eyelashes
knuckles
back
seeds
hair
stripes
ground/grass
horns
red
moustache
grey
plants
rectangular
blue
black
hairy
torso
shadow
tree
roof
leaves
neck
fur
thumb
metallic
coiled
feet
brown
tail
brick
curved
shoes
flesh
beard
tower
arm
nail
stubble
white
hooves
dress
dimples
blonde
collar
straight
cheeks
ear
eye
furry
glasses
lips
nostrils
shirt
teeth
wall/door
forehead/eyebrow

IT
categories

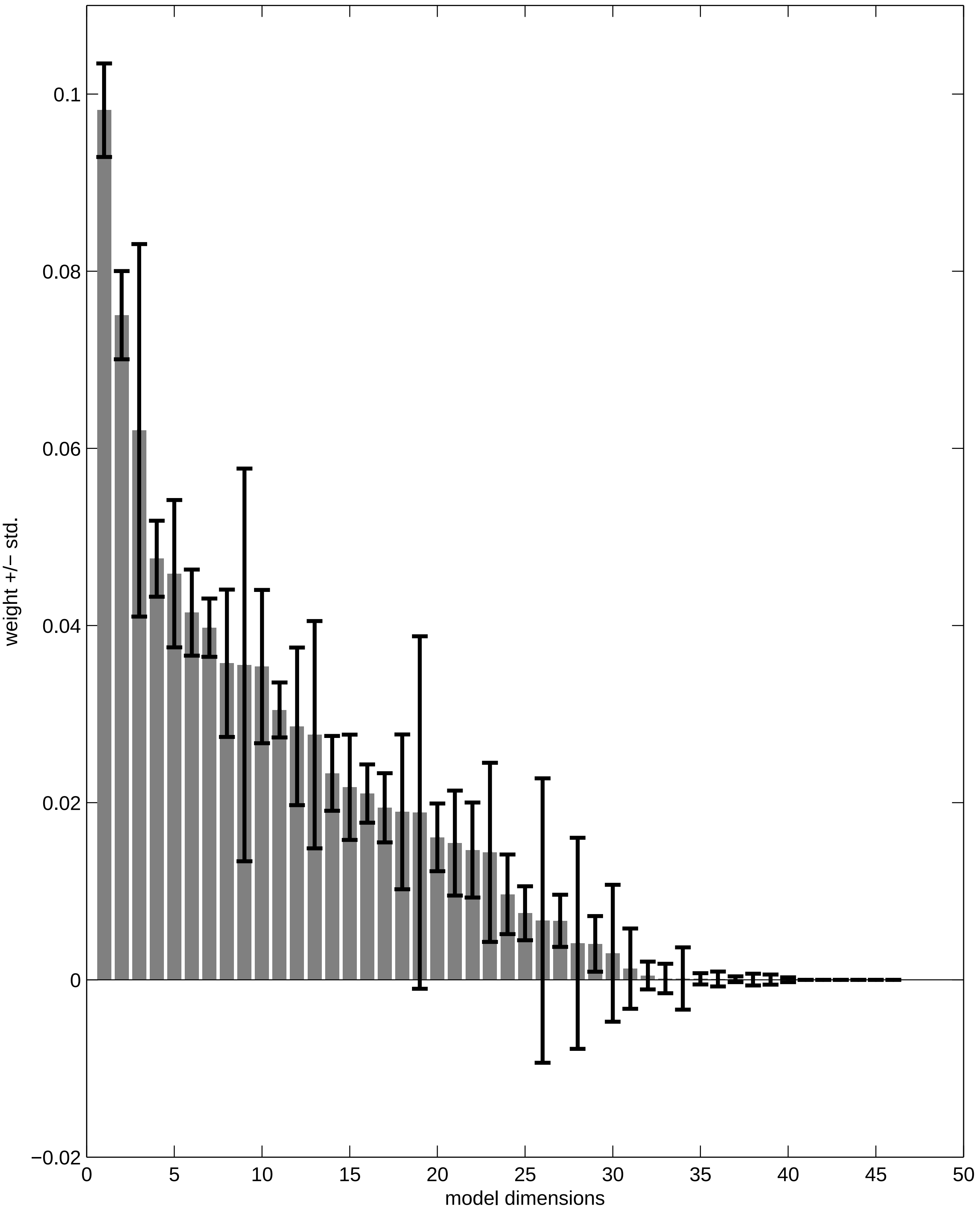

IT
categories

landscape
tree
hand
baboon
structure/architecture
organism/living
adult/human/person
building
pepper
urban
face
technology
sign/road sign
mammal
vertebrate
nonliving/manmade
woman/female
tool/equipment
gesture
fruit
canine/dog/pet
snake
symbol
vegetable
man
sense
food/edible
warning
monkey
body part
reptile
primate
cold−blooded
livestock
male
salad
house/dwelling
carnivore
herbivore
entrance
animal
artificial
horned
natural
object
shelter

SJ
features

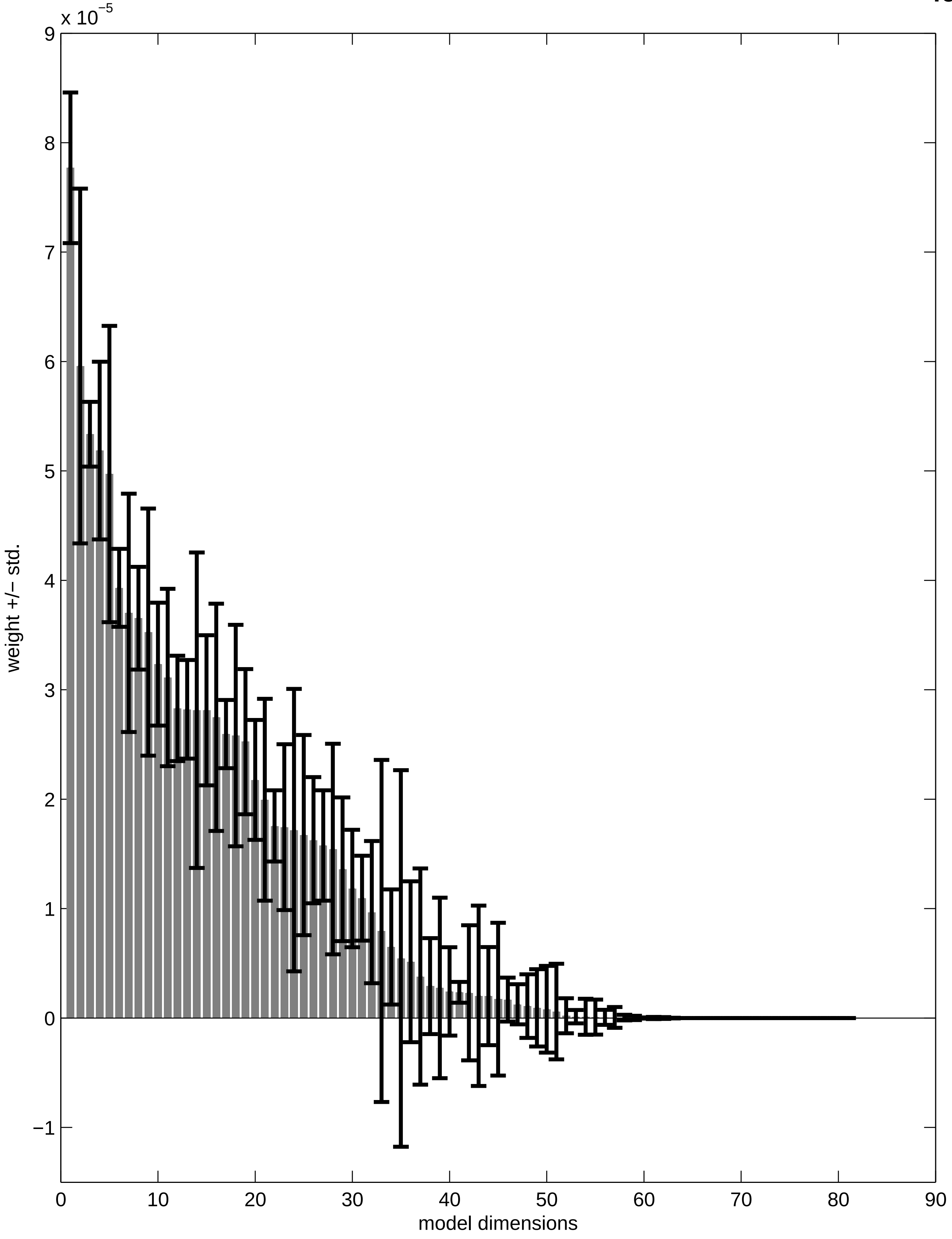
x 10^−5
9
8
7
6
5
4
3
2
1
0
−1
weight +/− std.
0
10
20
30
40
50
60
70
80
90
model dimensions

SJ
features

coiled
rectangular
head
yellow
window
skin
woolly
red
metallic
snout
arrow
green
sky
glass
shiny
hand/fingers
legs/body
handle
round/circular
leafy
wooden
furry
shadow
rounded
horns
symmetrical
stripes
building
curved
feet
brown
shoulder
plastic
tail
teeth
tower
knuckles
white
eyelashes
leaves
nail
grey
roof
seeds
wet/water
hair
blue
black
shoes
plants
thumb
straight
torso
brick
flesh
hooves
tree
hairy
back
wrist
fur
stubble
ground/grass
arm
beard
blonde
cheeks
collar
dimples
dress
ear
eye
glasses
lips
moustache
neck
nostrils
shirt
wall/door
forehead/eyebrow
nose/mouth

SJ
categories

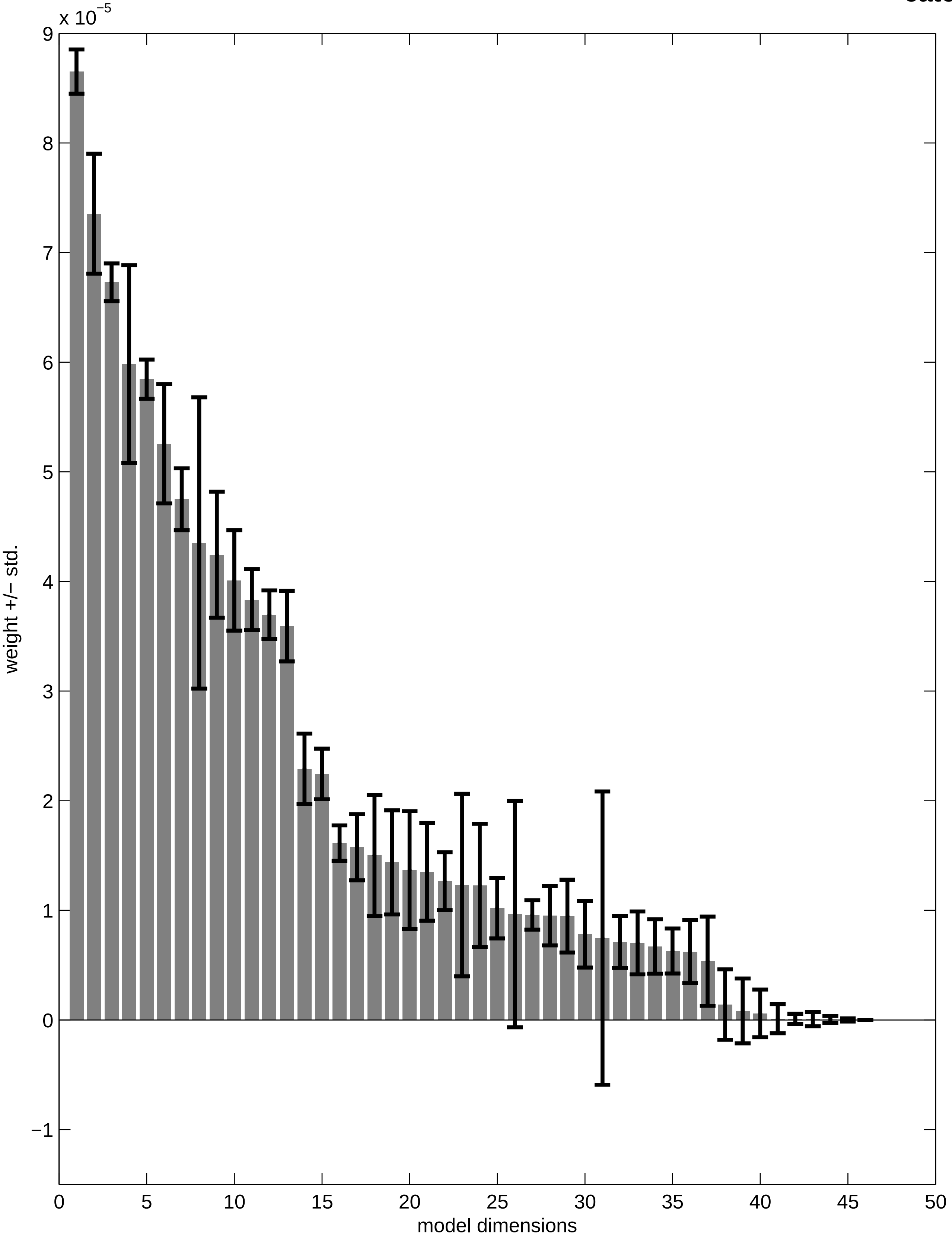

SJ
categories

food/edible
tree
landscape
structure/architecture
body part
cold−blooded
animal
tool/equipment
technology
symbol
nonliving/manmade
natural
organism/living
man
woman/female
vegetable
herbivore
livestock
snake
carnivore
canine/dog/pet
vertebrate
building
sign/road sign
adult/human/person
urban
fruit
object
hand
monkey
pepper
baboon
artificial
mammal
face
primate
sense
gesture
horned
warning
male
house/dwelling
shelter
reptile
entrance
salad

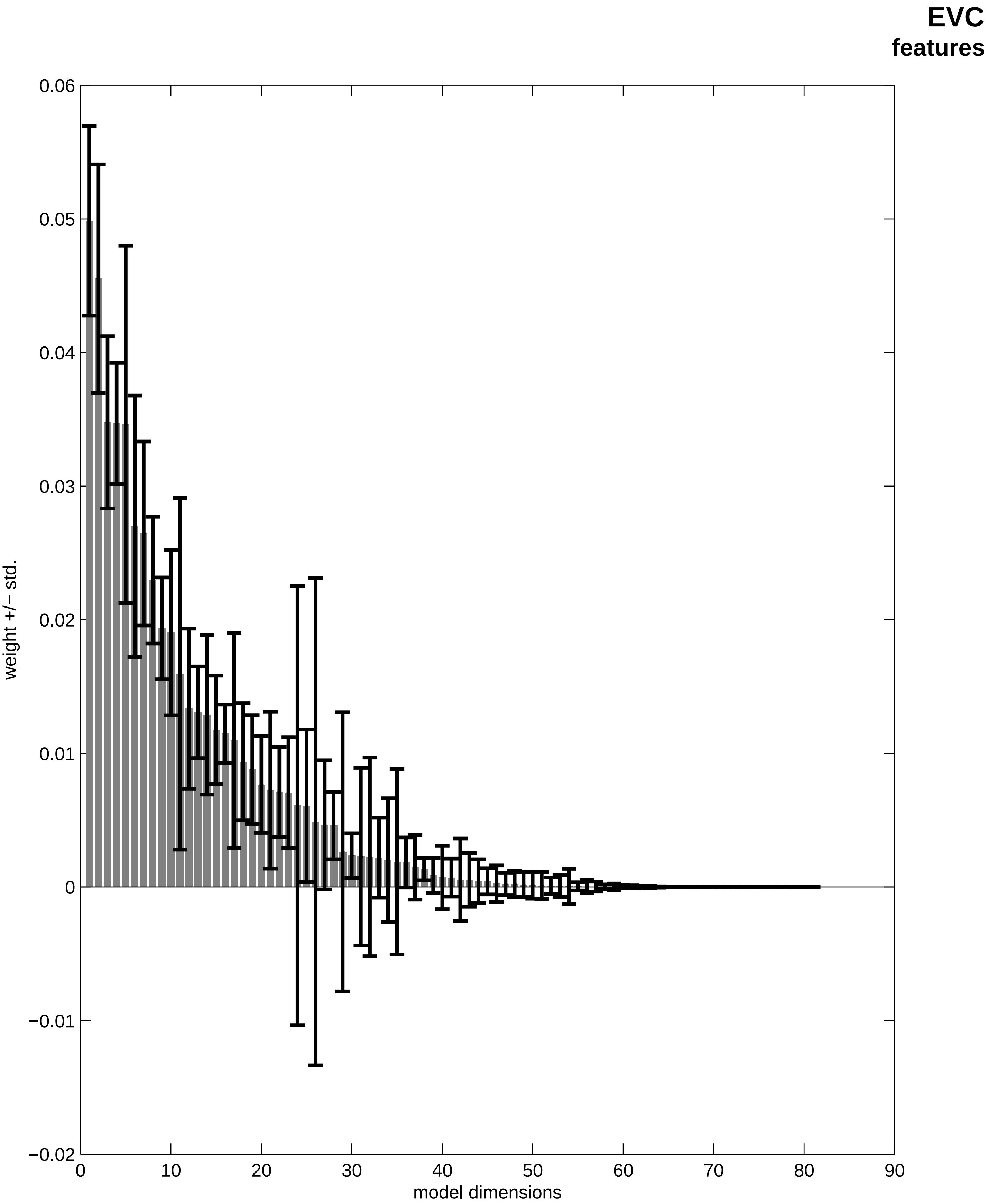

EVC
features
weight +/− std.
model dimensions
0.06
0.05
0.04
0.03
0.02
0.01
0
−0.01
−0.02
0
10
20
30
40
50
60
70
80
90

EVC
features

rounded
handle
shiny
snout
hand/fingers
woolly
shoulder
hairy
wooden
window
knuckles
feet
straight
wrist
tail
nose/mouth
eyelashes
yellow
arm
cheeks
dress
red
black
grey
metallic
torso
fur
green
thumb
skin
glass
rectangular
curved
shoes
wet/water
eye
symmetrical
head
neck
back
furry
plastic
arrow
blonde
nostrils
dimples
leafy
stubble
seeds
brick
hooves
teeth
stripes
flesh
roof
glasses
moustache
hair
nail
white
wall/door
collar
horns
brown
leaves
beard
blue
building
coiled
ear
lips
plants
shadow
shirt
sky
tower
tree
legs/body
round/circular
forehead/eyebrow
ground/grass

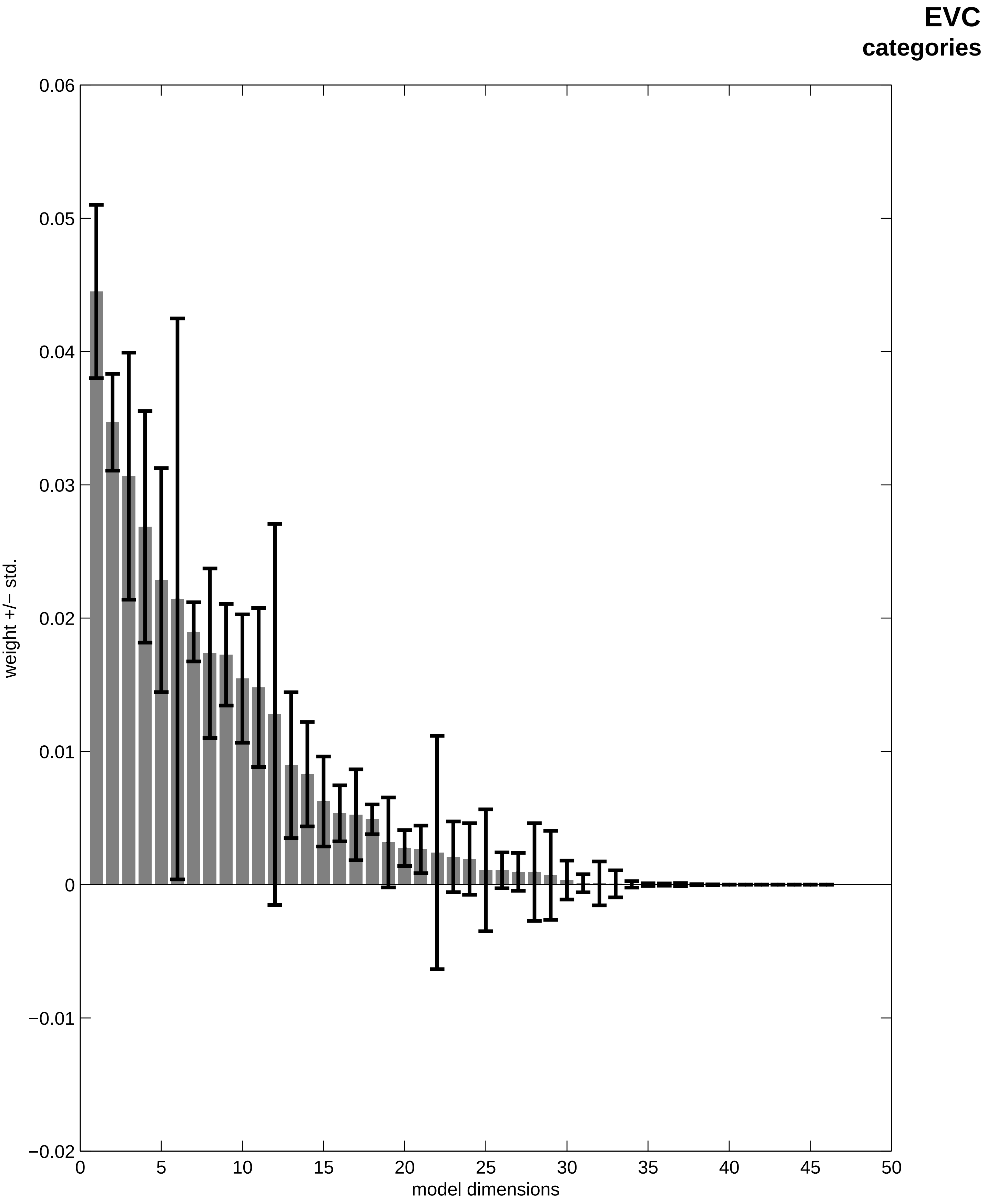
EVC
categories
weight +/− std.
model dimensions
0.06
0.05
0.04
0.03
0.02
0.01
0
−0.01
−0.02
0
5
10
15
20
25
30
35
40
45
50

EVC
categories

hand
woman/female
tool/equipment
baboon
warning
pepper
face
primate
man
canine/dog/pet
gesture
sense
monkey
house/dwelling
horned
adult/human/person
shelter
nonliving/manmade
structure/architecture
mammal
object
technology
herbivore
building
landscape
artificial
body part
symbol
salad
urban
male
livestock
cold−blooded
tree
organism/living
vertebrate
vegetable
fruit
entrance
animal
carnivore
natural
reptile
snake
food/edible
sign/road sign
